# Supplementary material for: The speed of GTP hydrolysis determines GTP cap size and controls microtubule stability
Source: eLife. 2020 Feb 13;9:e51992. doi: 10.7554/eLife.51992 (PMC7018511; doi:10.7554/eLife.51992)
Supplement: Transparent reporting form [file elife-51992-transrepform.docx]

***eLife’s* transparent reporting form**

We encourage authors to provide detailed information *within their submission* to facilitate the interpretation and replication of experiments. Authors can upload supporting documentation to indicate the use of appropriate reporting guidelines for health-related research (see [EQUATOR Network](http://www.equator-network.org/%20)), life science research (see the [BioSharing Information Resource](https://biosharing.org/)), or the [ARRIVE guidelines](http://www.plosbiology.org/article/info:doi/10.1371/journal.pbio.1000412) for reporting work involving animal research. Where applicable, authors should refer to any relevant reporting standards documents in this form.

If you have any questions, please consult our Journal Policies and/or contact us: [editorial@elifesciences.org](mailto:editorial@elifesciences.org).

**Sample-size estimation**

- You should state whether an appropriate sample size was computed when the study was being designed
- You should state the statistical method of sample size computation and any required assumptions
- If no explicit power analysis was used, you should describe how you decided what sample (replicate) size (number) to use

Please outline where this information can be found within the submission (e.g., sections or figure legends), or explain why this information doesn’t apply to your submission:

No statistical method was used to predetermine sample size. Based on our experience and other previously reported data, the number of analyzed microtubules, EB3 comets and single molecules is high and can be considered robust to detect and measure clear differences (and/or lack of differences) between conditions.

**Replicates**

- You should report how often each experiment was performed
- You should include a definition of biological versus technical replication
- The data obtained should be provided and sufficient information should be provided to indicate the number of independent biological and/or technical replicates
- If you encountered any outliers, you should describe how these were handled
- Criteria for exclusion/inclusion of data should be clearly stated
- High-throughput sequence data should be uploaded before submission, with a private link for reviewers provided (these are available from both GEO and ArrayExpress)

Please outline where this information can be found within the submission (e.g., sections or figure legends), or explain why this information doesn’t apply to your submission:

HPLC experiments for nucleotide content determination were performed three times. All the numbers for microscopy experiments (averaged microtubules, microtubule ends, single molecules, and time-lapse frames analyzed or averaged for each experiment) are always stated in the respective figure legends. Microtubule growth speed and comet shape measurements pool data from several independent experiments or replicates. EB3 Kd measurements for WT and mutant microtubules are from one experimental replicate with high numbers of microtubules measured for each EB3 concentration. Supplementary experiment with GMPCPP bound WT recombinant microtubules and to demonstrate a lack of EB3 binding was performed once – the non-binding was uniform across thousands of microtubules in the sample. The same results are consistent with the published literature as well as numerous independent examples of the lack of EB3-binding to GMPCPP-stabilized pig brain microtubules throughout the paper. The experiment with E254A microtubules and GMPCPP-bound pig brain microtubules to test TPX2micro binding was performed once. Again, TPX2micro was always highly bound to thousands of GMPCPP microtubules and faintly bound to thousands of E254A microtubules in the sample. The experiment where E254A tubulin was buffer exchanged into GMPCPP-contained buffer and then polymerized in the presence of GMPCPP, was performed twice with similar results. Images from independent experiments are presented on the supplementary figure. No data were excluded except for obvious reasons (e.g. sample not in focus, microtubule growing out of the field of view, crossing over other microtubules).

**Statistical reporting**

- Statistical analysis methods should be described and justified
- Raw data should be presented in figures whenever informative to do so (typically when N per group is less than 10)
- For each experiment, you should identify the statistical tests used, exact values of N, definitions of center, methods of multiple test correction, and dispersion and precision measures (e.g., mean, median, SD, SEM, confidence intervals; and, for the major substantive results, a measure of effect size (e.g., Pearson's r, Cohen's d)
- Report exact p-values wherever possible alongside the summary statistics and 95% confidence intervals. These should be reported for all key questions and not only when the p-value is less than 0.05.

Please outline where this information can be found within the submission (e.g., sections or figure legends), or explain why this information doesn’t apply to your submission:

The analysis methods are described in detail in materials and methods. Representative raw data is presented as microscopy images, HPLC profiles and protein gels and described in detail in the figure legends. Sample size, detailed information of the plot content and precision measures (mean, SD or SEM) are also stated in the figure legends. P-value calculations were not applied in this study.

(For large datasets, or papers with a very large number of statistical tests, you may upload a single table file with tests, Ns, etc., with reference to sections in the manuscript.)

**Group allocation**

- Indicate how samples were allocated into experimental groups (in the case of clinical studies, please specify allocation to treatment method); if randomization was used, please also state if restricted randomization was applied
- Indicate if masking was used during group allocation, data collection and/or data analysis

Please outline where this information can be found within the submission (e.g., sections or figure legends), or explain why this information doesn’t apply to your submission:

The study is centered around the analysis of three different tubulin species (wt and two mutants). The experiments include comparisons of different biological aspects between these three tubulins. Randomization/blinding was not applicable nor was it possible - the three tubulin species studies here showed distinct and obvious phenotypes in all experiments.

**Additional data files (“source data”)**

- We encourage you to upload relevant additional data files, such as numerical data that are represented as a graph in a figure, or as a summary table
- Where provided, these should be in the most useful format, and they can be uploaded as “Source data” files linked to a main figure or table
- Include model definition files including the full list of parameters used
- Include code used for data analysis (e.g., R, MatLab)
- Avoid stating that data files are “available upon request”

Please indicate the figures or tables for which source data files have been provided:

N/A
